# A Water-Damaged Home and Health of Occupants: A Case Study

**DOI:** 10.1155/2012/312836

**Published:** 2011-12-15

**Authors:** Jack Dwayne Thrasher, Michael R. Gray, Kaye H. Kilburn, Donald P. Dennis, Archie Yu

**Affiliations:** ^1^Citrus Heights, CA, USA; ^2^Progressive Healthcare Group, Benson, AZ 85602, USA; ^3^Neurotest, Inc., Pasadena, CA 91107, USA; ^4^USC Keck School of Medicine, Los Angeles, CA 90089, USA; ^5^Center for ENT and Facial Plastic Surgery, Atlanta, GA 30327, USA; ^6^Compliance Solution, Honolulu, HI 96823, USA

## Abstract

A family of five and pet dog who rented a water-damaged home and developed multiple health problems. The home was analyzed for species of mold and bacteria. The diagnostics included MRI for chronic sinusitis with ENT and sinus surgery, and neurological testing for neurocognitive deficits. Bulk samples from the home, tissue from the sinuses, urine, nasal secretions, placenta, umbilical cord, and breast milk were tested for the presence of trichothecenes, aflatoxins, and Ochratoxin A. The family had the following diagnosed conditions: chronic sinusitis, neurological deficits, coughing with wheeze, nose bleeds, and fatigue among other symptoms. An infant was born with a total body flare, developed multiple Cafe-au-Lait pigmented skin spots and diagnoses with NF1 at age 2. The mycotoxins were detected in bulk samples, urine and nasal secretions, breast milk, placenta, and umbilical cord. *Pseudomonas aueroginosa, Acinetobacter, Penicillium,* and *Aspergillus fumigatus* were cultured from nasal secretions (father and daughter). RT-PCR revealed *A. fumigatus* DNA in sinus tissues of the daughter. The dog had 72 skin lesions (sebaceous glands and lipomas) from which trichothecenes and ochratoxin A. were detected. The health of the family is discussed in relation to the most recent published literature regarding microbial contamination and toxic by-products present in water-damaged buildings.

## 1. Introduction

Indoor dampness and fungal contamination have been shown in qualitative reviews to be associated with a variety of respiratory health effects, including infections, sinusitis, and otitis media [1–4]. In addition, case studies with and without controls have demonstrated the existence of severe sinusitis as well as neurological deficits in occupants in water-damaged homes and buildings [5–12]. Currently, it is recognized that the indoor water-damaged environment resulting from microbial growth is a complex mixture of mold and bacteria along with their by-products [13–15]. Thus, the illnesses resulting from exposure cannot be defined by any specific component of the affected environment [2, 13–17]. In this paper we present a family of five exposed to fungi and bacteria in a water-damaged home located in Maui, Hawaii. Members of the family developed multiple health problems, including sinusitis and neurological deficits. In addition, the mother was pregnant during occupation of the contaminated home giving birth to a girl who had a total body flare with development of Cafe-au-Lait spots. Her condition has been diagnosed with Neurofibromastosis type (NF1).

## 2. The Family

**Figure 1:**
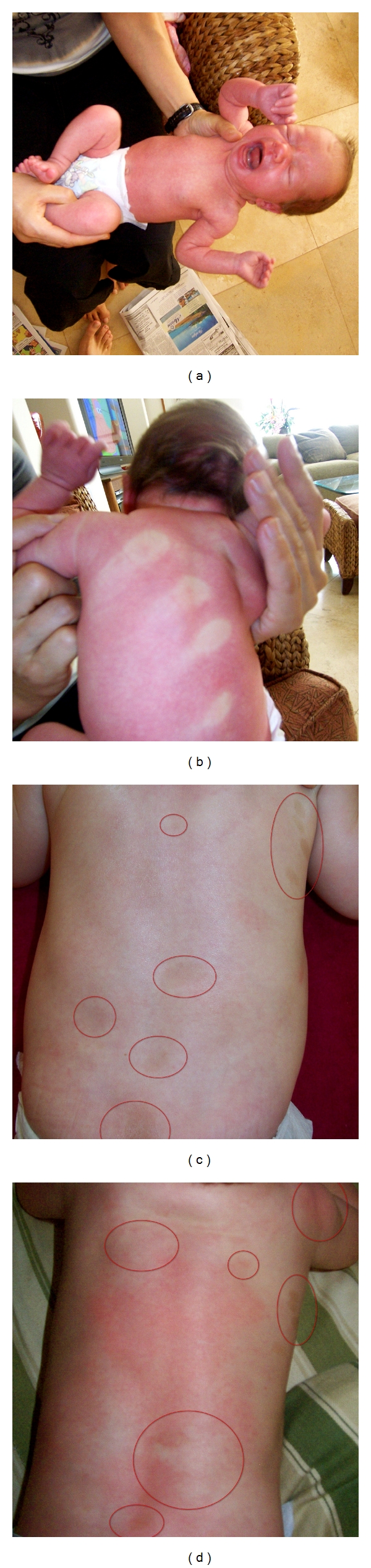
The upper two photos are of the newborn girl demonstrating the total body flare and the impression of the Father's hand on her back. The bottom two photos show the pigmented sports that appear to be Cafe-au-Lait skin pigmentation that were apparent at birth and are still present. The flare reaction was present at birth, began to subside at 10–12 weeks, and occurred periodically through 55 weeks of age. The multiple pigmented spots has been diagnosed as NF1 at U.S. San Francisco, Department of Dermatology.

**Figure 2:**
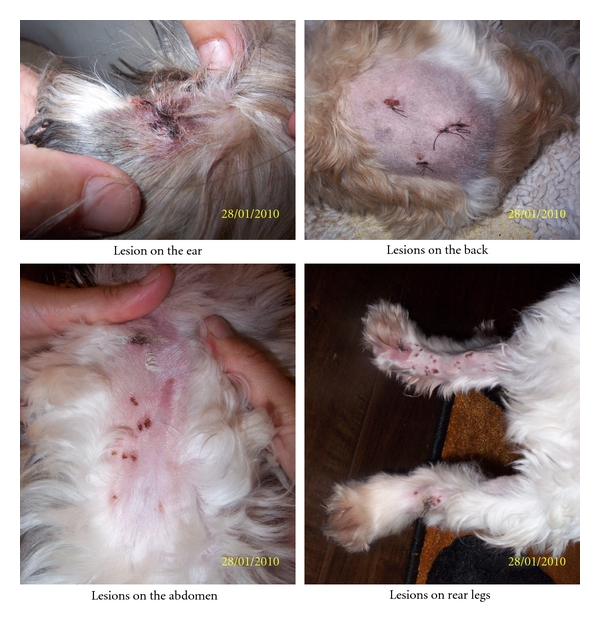
This figure demonstrates the sites of the subcutaneous and lipoma tumors that were removed from the pet dog. The Veterinarian stated that the presence of 72 such lesions on an animal is a very rare observation.

The family of five moved from Canada to Maui, Hawaii, in February 2008, where they rented a home. All were healthy prior to the move and began experiencing symptoms shortly after the move in. Chief health complaints were as follows. Father (age 40) had persistent cough with phlegm, throat irritation, headaches, sinusitis, severe fatigue, somnolence, decreased concentration, long-term and recent memory loss, nose bleeds, decreased libido, hair loss, and shortness of breath with wheezing. The mother (age 39) complained of cough with phlegm, throat irritation, headaches, sinusitis, extreme fatigue, somnolence, recent and long-term memory loss, decreased libido, and shortness of breath with wheezing. She became pregnant while living in the home and gave birth to a girl 3 months after moving out of the home. The eldest daughter (age 8) had the same symptoms as the parents, except she had decreased concentration, nausea, and loss of appetite. The son (age 5) had frequent headaches, fatigue and tiredness, nasal congestion, nose bleeds, throat irritation, shortness of breath with mild wheezing, and decreased attention in classroom activities. The newborn had a total body flare (pinkish red) that continued to age 10–12 weeks, after which the flare would appear periodically. She had multiple pigmented skin spots on her back, chest, and abdomen at birth that appeared to be Cafe-au-Lait spots. The pigmented areas are still present at 2 years of that are scheduled for additional diagnostics for neurofibromatosis (Figure 1). Finally, the pet dog developed approximately 72 skin lesions diagnosed as sebaceous and lipoma tumors (Figure 2).

## 3. Neurological Evaluation

The family sought neurological consultation from one of the authors as previously published [8, 9]. The results of the evaluations are briefly summarized as follows.

The father had 17 neurological deficits as follows: simple and choice reaction time, sway-balance with eyes open and closed, decreased right and left grip strength, abnormal right and left color vision, abnormal visual field performance (right and left), abnormal digit symbol, abnormal perceptual motor speed (dominant pegboard, Trails A and B, right and left finger writing errors), abnormal smell score, abnormal picture completion and elevated Profile of Mood States (POMS), Beck's depression inventory, and Limbic System Check List score. The increased POMS score was consistent with elevated confusion, fatigue, and tension. The mother also had 17 abnormalities, identical to those of the husband (data not repeated). The neurological scores for the daughter were within normal ranges. However, the physical exam revealed abnormal past pointing without dysmetria (finger to nose) and fine resting tremors at 3-4 per second increasing to 10 by intention with amplitude increased. The son (age 5) did not have any detectable neurological deficits. However, the neurological testing is not designed for 5 year olds.

In conclusion, the neurological evaluation revealed multiple deficits in both parents as previously published [8, 9]. The daughter had noticeable tremors which may have resulted from exposure to tremorgenic mycotoxins [18–22] as well as others described here in after (see Section 9 and Tables 4 and 5).

## 4. MRI

MRIs were performed at Oak Tree Medical Imaging, Pasadena, California, for each family member with special reference to the sinuses.

**Father**

The father had mild diffuse thickening- bi-ethmoid, bi-maxillary, right sphenoid and frontal sinuses.

**Mother**

The cavernous and paranasal sinuses were normal. Prior to the MRI, she had been prescribed corticosteroids, antibiotics, and antifungals.

**Daughter**

The daughter had mild fluid within the bilateral mastoid air cells. There is moderate to severe mucosal thickening in the maxillary and ethmoid sinuses without evidence of air fluid level.

**Son**

The bifrontal and sphenoid sinuses have not developed. Maxillary sinuses are unremarkable. There is slight mucosal thickening within the bilateral sphenoid sinuses, right greater than left without air fluid level.

In conclusion, the results of the MRI studies demonstrated mucosal thickening of the sinuses of the father and two children. The absence of findings in the mother most likely resulted from the use of corticosteroids and medications to treat her sinusitis.

## 5. ENT Evaluation

The father and daughter were evaluated at the Atlanta Center for ENT & Facial Plastic surgery according to procedures previously published [5, 6]. The results of the evaluation are briefly summarized as follows.

**Father**

Nasal endoscopy revealed (a) nasal polyps and (b) the ethmoid, sphenoid, and frontal sinuses were edematous with visible thick mucoid material (mucin) bilaterally, confirming the results of earlier MRI and CT scans (data not described). Total IgE was 76.9 IU/mL with a positive IgE score at level IV for *Alternaria*. He was tested for IgG antibodies for ten fungi and was positive for* Epiccocum *and* Cladosporium* at level I, *Penicillium, Aspergillus, Alternaria, Fusarium, *and* Acremonium* at level III, and *Candida* at level III. Recommended treatment was saline nasal wash, intranasal amphotericin B, oral fluconazole, Nystatin, intranasal glutathione, and oxygen via a face mask. Surgery was performed to remove nasal polyps and inflamed sinus tissues. Tissue samples were sent to RealTime Laboratories, Carrollton, Texas, for RT-PCR DNA probes (10 species of fungi), and mycotoxin testing.

The RT-PCR-DNA probes were negative for the following fungi: *Aspergillus flavus, fumigatus, niger, *and *versicolor; Eurotium amstelodami; Fusarium solani; Penicillium chrysogenum *and* verrucosum; *and* Stachybotrys chartarum* and* echinata*. Cultures for bacteria (SBA) and fungi (MEA) in nasal secretions were positive for *Pseudomonas aeurogino*sa and *Penicillium spp*.

**Daughter**

Endoscopic examination revealed that left maxillary, ethmoid, sphenoid, and frontal recesses were edematous. The turbinates were 4+ enlarged. The nasal septum was deviated to the left. On the right side there was some white material on the middle turbinate. The adenoids were hypertrophied. In addition, small white flecks were present in the soft tissue of the left maxillary, ethmoid, and left sphenoid sinuses. Medications include fluconazole, liposomal glutathione, amphotericin B, inhaled corticosteroid, Nystatin, and oxygen via face mask. The patient required left sphenoidotomy. Also, the previous MRI and CT scans showed opacification of the left infundibulum and left maxillary sinus os. Surgical specimens were sent to RealTime Laboratories tor RT-PCR DNA probe (10 species of fungi) and mycotoxin detection.

The RT-PCR tests were negative for the same species as done on the father (see above). However, cultures for bacteria (SBA) and molds (MEA) on nasal secretions revealed *Acinetobacter spp*. and *Aspergillus fumigatus*.

In conclusion, the nasal endoscopic examinations of the father and daughter revealed edematous inflammation of the paranasal sinuses that required surgery. The RT-PCR tests were negative for 10 species of fungi, which did not eliminate the presence of fungi other than those tested. Finally, bacterial and fungal cultures of nasal mucous secretions did reveal the presence of bacteria (*Pseudomonas *and *Acinetobacter*) as well as fungi (*Penicillium* and *Aspergillus*). Thus both patients had severe chronic rhinosinusitis most likely related to microbes (bacteria and fungi) detected in their water-damaged home [1–3, 5, 6, 23–25].

## 6. The Home

The home was inspected for construction defects and dampness by two independent services: Barkman Inspection Services [26] and Engineering Dynamics Corp [27]. The results of the two inspections are briefly summarized.

### 6.1. Barkman Report

A serious moisture/mold problem is observed in the crawlspace directly below the bedrooms. Moisture is penetrating the walls of the foundation. The HVAC system is designed to force air into the crawl space, forcing crawl space air into the bedrooms and other areas above. Moisture intrusion also results from the master shower into the crawl space as well as from sprinklers, damp soil against the foundation, lack of roof gutters, and poor grading.

### 6.2. Engineering Dynamics Report

This is a two-story house with a crawl space. Lower level has a family room, guest bedroom, bathroom, powder room, arts and crafts room, storage closet, garage, and crawl space, which are under upper level bedrooms and bathrooms. Upper level has 3 bedrooms, 3 bathrooms, entertainment room, living room, kitchen, office, and powder room.

The crawl space had water intrusion, musty mold odor, and visible mold on floor joists. The yard sprinklers were directed towards the house and the eaves did not have rain gutters, permitting the pooling of water. Water entered the crawl space through cement walls and followed piping present in the crawl space. Smoke testing revealed communication between the crawl space and upper level bedrooms via electrical outlets and electrical ducts and plumbing. The conduit holes were not sealed, permitting observance of light coming through spaces in the floor joists. A musty odor was present in the master bathroom and noted to get stronger when the fan coil was turned on.

## 7. Identification of Mold

All air and bulk samples were sent under chain of custody to EMSL Analytical, Inc., Westmont, NJ. The ERMI Q-PCR 36 for mold species was performed on 5 different bulk samples. The data are summarized in Table 1. The identified species of mold varied according to source but included species of *Aspergillus, Penicillium, Eurotium amstelodami, A pullulans, C. globosum*, and *T. viride*, among others. The ERMI interpretation level ranged from 2 to 3, indicating moderate contamination.

Airborne viable spores were determined by Air-O-Cell cassettes and cultured and identified by EMSL Method M050 and the data are summarized in Table 2. The viable airborne spores (Table 2) showed the presence of toxic fungi inside of the home and none outdoors. The viable spores included species of *Aspergillus* and *Penicillium*, which varied according to the sample area, for example, crawl space versus bedroom air and wall space cavity.

In conclusion, these data demonstrated that testing for fungal contamination must include several different sample locations involving dust and bulk materials as well as airborne viable spores [28].

## 8. Identification of Bacteria and Endotoxins

Bulk samples of crawl space dirt, gravel, plastic sheeting, wood, and a sandal from under the master bed were sent to EMSL Analytical, Inc., Westmont, NJ and RealTime Laboratories, Carrolton, TX, to culture and identify bacteria using sheep blood agar (SBA) plates. In addition, two swab samples from the kitchen were analyzed for endotoxins by EMSL. The results are summarized in Table 3.

Bacteria detected by both laboratories included Gram negative and positive organisms. The primary Gram positive bacteria included *Bacillus spp*, *Actinomycetes* (e.g., *Streptomyces *sp., *Mycobacterium hominis*), and *Staphylococcus* (non aureus). The Gram negative bacteria were species of* Pseudomonas* and* Proteus spp.* Both groups of bacteria are potential human pathogens. For example, *Mycobacterium *and *Streptomyces spp.* are capable of causing lung abscesses and granulomatous mycetomas, while* Pseudomonas *species can cause respiratory and other infections [29–31]. 

Endotoxins were tested in only two areas of the home. The J-tube under the kitchen sink, a relatively protected area, had a concentration of 4.930 EU per swab. In contrast, the top of the kitchen cabinet had a concentration of 24.800 EU/swab. The two control swabs were negative. These observations indicate that additional testing was probably warranted, since endotoxins cause respiratory inflammation, sensitizers, and exacerbation of asthma [32–35]. In conclusion, bacterial cultures identified potentially pathogenic Gram negative and positive bacteria. In addition, these bacteria are known to produce toxic secondary metabolites of which Valinomycin is a mitochondrial toxin and is synergistic with macrocyclic trichothecenes [36–39]. Recently, several toxic bacterial metabolites have been demonstrated to cooccur with mycotoxins in moisture-damaged indoor environments [15].

## 9. Identification of Mycotoxins in Environmental Samples and Body Fluids

Bulk samples were sent to RealTime Laboratories, Carrollton, TX, to test for the presence of mycotoxins. In addition, urine and nasal mucous were collected in sterile cups, sealed and sent to RealTime Laboratories to test for the presence of mycotoxins. The tests for macrocyclic trichothecenes, aflatoxins, and ochratoxin A were performed as previously reported [40].

### 9.1. Environmental Samples

The data for mycotoxins detected in bulk samples are summarized in Table 4. Trichothecenes and ochratoxin A were detected in the bathroom towel (11.71 and 4.9 ppb), respectively, and the sandal (0.47 and 3.4 ppb), respectively. Mycotoxins were identified in the samples from the crawl space as follows: Wood truss: trichothecenes (1.69 ppb), aflatoxins (3.5 ppb), ochratoxin A (5.8 ppb); Gravel: trichothecenes (7.7 ppb), ochratoxin A (7.7 ppb); Dirt: trichothecenes (2.1 ppb), ochratoxin A (2.1 ppb); and Plastic sheeting: ochratoxin A (2.8 ppb).

### 9.2. Body Fluids

Mycotoxins detected in body fluids of family members and the pet dog are summarized in Table 5. The father was positive for ochratoxin A in his urine (18.2 ppb), while two separate nasal mucous samples were positive for both aflatoxins (0.5 and 11.2 ppb) and ochratoxin A (18.2 ppb). The mother's urine contained ochratoxin A (18.2 ppb), while nasal mucous contained the three mycotoxins aflatoxin, ochratoxin A, and trichothecenes at 1.02, 1.2, and 1.5 ppb, respectively. The daughter's urine had trichothecenes (0.23 ppb) and ochratoxin (28 ppb), while nasal mucosa had trichothcenes (4.68 ppb) and ochratoxin A (3.8 ppb). The urine sample from the son was positive for ochratoxin A (18.9 ppb), while tests on nasal mucous were not performed. The urine from the pet dog was positive for trichothecenes (1.49 ppb) and ochratoxin A (25.9 ppb).

## 10. Newborn Baby

The mother gave birth to a girl who was born with a total body flare 3 months after vacating the home (Figure 1). The infant was born with pigmented skin identified as Cafe-au-lait. They are currently distributed as follows: Face (2), neck (6), right axilla (9), left axilla (10), left and right arms (4), abdomen (16), back (28), buttocks (9), right leg (8), and left leg (2) for a total of 84. As a result, breast milk, placenta, umbilical cord, and the baby's urine were tested for the presence of mycotoxins. Ochratoxin A was detected in the breast milk (2.7 ppb), placenta (4.2 ppb), and the umbilical cord (7 ppb). The newborn's urine was negative for mycotoxins. In retrospect, the amniotic fluid (lost during birth) should have been tested.

## 11. Pet Dog

The pet dog had approximately 72 skin lesions on its legs, trunk, and ears (Figure 2). The lesions were surgically removed. Pathology of the ear mass described it as a sebaceous gland, while the other lesions were lipomas. Tests for mycotoxins in the surgical specimens revealed the following: Ear mass—trichothecenes (23.07 ppb) and ochratoxin A (2.2 ppb); and Lipoma—trichothecenes (20.9 ppb) and ochratoxin A (1.4 ppb). The veterinarian stated that lipomas in dogs are normal; however, the presence of multiple lipomas is a rare occurrence.

## 12. Discussion

We have presented a family of five who had no history of health problems until they moved into a water-damaged home in Hawaii. Shortly after the move in they began to develop multiple symptoms, sought medical consultation for the health problems involving the upper and lower respiratory tract, headaches, neurocognitive deficits, and severe sinusitis. Neurological evaluation revealed 17 areas of neurological abnormalities in the two adults, consistent with previous reports [8, 9]. The daughter developed tremors that could be related to exposure to tremorgenic and other mycotoxins [18–22]. The son, age 5 at the time of examination, did not have neurological deficits. However, he did have a variety of symptoms (e.g., nose bleeds, cough, wheeze, and headaches) consistent with exposure to water-damaged indoor environments. In addition, when he began school, the teacher reported lack of concentration while in class. Perhaps he was showing signs of autistic spectrum disorder and/or ADD/ADHD as previously reported in children exposed to water-damaged home environments [10].

The parents and the two children have chronic sinusitis and nasal inflammation. The isolation of bacteria (*Pseudomonas *and *Acinetobacter*) and molds (*Penicillium *and *Aspergillus*) from nasal secretions from the father and daughter is consistent with the literature. Bacterial and fungal sinusitis has been reported [1, 5, 6, 23–25]. In addition, the detection of mycotoxins in the nasal secretions from the family points towards fungal rhinosinusitis. Finally, the culture of surgical specimens taken from the daughter's sphenoid/ethmoid mucosa identified* Aspergillus fumigatus*.

Macrocyclic trichothecenes and tremorgens have been detected in airborne fungal fragments less than the size of conidia [22, 41–43]. Furthermore, trichothecenes, aflatoxins, sterigmatocystin, ochratoxin A, and other mycotoxins are present in the dust of water-damaged buildings [13, 16]. In addition, indoor microbial growth fragments, releasing particulates less than one micron that penetrate deep into the alveolar spaces [44–46]. Thus, the presence of trichothecenes, ochratoxin A, and aflatoxins in bulk samples (Table 4) and body fluids of the family (Table 5) is interpreted as an inhalation exposure resulting in uptake of mycotoxins attached to dust and fine microbial particulates. Moreover, it is reported in this issue and elsewhere that these mycotoxins are present in the urine and tissue biopsy/necropsy materials taken from individuals residing in water-damaged homes and buildings [42, 47–50].

The newborn girl had a total body flare at birth that began to clear at 10–12weeks after birth, which may have been associated with mast cell/eosinophil activity. However, medical workup was not done in this area. The body flaring periodically appeared until approximately 55 months of age. The majority of the Cafe-au-Lait spots were apparent soon after delivery and continued to develop after birth and continue to be present (Figure 1). She was diagnosed with NF1 by Dr. Frieden at U.C.S.F. at age 2, and additional diagnostics are anticipated. The placenta, umbilical, breast milk, urine, and nasal secretion of the mother were positive for Ochratoxin A (Table 5), while a urine sample from the infant was negative. It is reasoned that amniotic fluid (lost at birth) would have been a better choice for mycotoxin testing. However, the presence of ochratoxin A in the placenta and umbilical cord suggests that the infant most likely was exposed in utero. There is no family history of NF1 leading Dr. Frieden with conclusion that the mutation to NF1 gene most likely occurred sometime during in utero development. It is possible that her condition could be related to ochratoxin A or other toxins known to be present in water-damaged buildings.

A few comments are in order regarding the pet dog. The dog developed 72 cutaneous lesions that were distributed over its body, including the ears (Figure 2). The dog's urine was positive for ochratoxin A and trichothecenes. In addition, surgical specimens of the ear (sebaceous gland) and body tumors (lipomas) were also positive for trichothecenes and ochratoxin A. The question that arises is were the growths caused by the mycotoxins or were they storage sites for the toxins.

In conclusion, a family of five (one *in utero*) was exposed to several species of mold and bacteria while occupying a water-damaged home. They presented with multiple symptoms, including chronic sinusitis, fatigue, and neurological complaints. Testing of the home revealed the presence of both mold and bacteria. Differential diagnostic procedures demonstrated in up to seventeen areas of central nervous system deficits as well as chronic fungal/bacterial sinusitis. 

 Mycotoxins testing demonstrated that ochratoxin A was the predominant mycotoxin in samples of urine, nasal secretions, breast milk, placenta, and umbilical cord. Lesser concentrations of macrocyclic trichothecenes were also detected. A newborn girl had a total body flare and had Cafe-au-Lait pigmentation spots. The infant is scheduled for further evaluation for her NF1 condition. This case study indicates that mold and bacteria and by-products in water-damaged homes are most likely the cause of the adverse health conditions of these occupants.

## Figures and Tables

**Table 1:** This table summarizes the results of the E.P.A. ERMI PCR-DNA tests performed on 5 mg dust samples from basement and master bedroom carpeting and master bedroom wall insulation. Only the species detected are listed.

Sample 36 ERMI Q-PCR test	Carpet basement	Carpet, master Bdrm	Insulation master Bdrm^1^	Insulation return air duct	Moist fiberglass
Group 1 Molds					
*Asp. penicillioides *	77	26	ND	ND	
*A. restrictus *	ND	ND	ND	40	40
A. versicolor	ND	ND	ND	ND	50
*E. amsteloda*mi	ND	ND	ND	4	4
*Aur. pullulans *	189	20	ND	ND	ND
*Ch. globosum *	ND	14	ND	ND	2
*Cl. Sphaerospermum *	9	3	ND	ND	ND
*Pae. variotii *	ND	2	87	ND	734
*P. brevicompactum *	ND	19	ND	ND	ND
*P. corylophilum *	ND	ND	ND	ND	85
*P. crustosum *	ND	ND	3	ND	ND
*P. purpurogenum *	ND	2	ND	ND	ND
*P. spinulosum *	15	ND	3	ND	ND
*P. variabile *	ND	ND	ND	136	3
*T. viride *	ND	ND	NS	ND	15
Sum of the Logs	6.6	6.2	2.8	2.8	10.6

Group 2 Molds					
*A. ustus *	2	4	187	ND	226
*Cl. cladosporioides *II	1	ND	ND	65	2
*Ep. nigrum *	15	17	ND	65	8
*Ep. nigrum *	15	17	ND	14	5
*Mucor/Rhizopus *	9	21	ND	ND	ND
*P. chrysogenum *	5	4	8.738	ND	14.013
Sum of the logs	3.3	3.7	6.2	3.0	8.1

ERMI Value	3	2	−3	0	3

ERMI Interpretation	Level 3	Level 3	Level 2	Level 2	Level 3

ND: Not detected.

^1^RT-PCR detected *Aspergillus fumigatus* in a towel taken from the master bathroom.

All values are in Spores E.−/mg dust.

**Table 2:** This table summarizes the identification and enumeration of culturable air-borne fungi collected by Aerotech cassettes (including speciation of *Penicillium*, *Aspergillus, Cladosporium, and Stachybotrys*) by EMSL Method M050.

Sample location	Media	Temp (°C)	Sensitivity & dilution	Fungal identification	Colon count	CFU per cassette
Swimming pool deck	MEA	25	100 & 100	None detected	0	0

Master bedroom	MEA	25	100 & 100	*Asp. sydowii *	1	100
			100 & 100	*Cl. sphaerospermum *	1	100
				*P. chrysogenum *	1	100
				**Total**	**3**	**300**

Crawl space	MEA	25	100 & 100	*Asp. ochraceus *	5	500
			100 & 100	*Asp. sydowii *	2	200
			100 & 100	*P. chrysogenum *	1	100
			1000 & 1000	*P. citreonigrum *	1	1000
			1000 & 1000	*Phialophora sp. *	1	1000
			1000 & 1000	Sterile (dark) sp.	1	1000
				**Total**	**11**	**3.800**

Wall space master bedroom	MEA	25	100 & 100	*Asp. fumigatus *	1	100
			100 & 100	*Asp. ustus *	3	300
			100 & 100	*Paecilomyces sp. *	2	200
			1000 & 1000	*P. chrysogenum *	1	1000
				**Total**	**7**	**1.600**

**Table:** (a)

Sample	Sample #	Media	Temp (°C)	Analytical sensitivity CFU/g	Bacteria	Colony count	CFU/g
Plastic sheeting, crawl space	#34	SBA	35	98.000	*Bacillus sp * *Streptomyces sp.* *Actinomycetes*	25	2.450.000

Moist gravel, crawl space	#27	SBA^1^	35	885	*B. megaterium * *Bacillus sp.* **Total**	10 7 **17**	8.850 6.190 **15.000**

Moist dirt, crawl space	#28	SBA^1^	35	8130	*B. megaterium * *Bacillus sp.* **Total**	4 6 **10**	32.500 48.800 **81.300**

Swab of wood, crawl space	#25	SBA^2^	35	10.000	*Microbacterium hominis * *Staphylococcus sp (not aureus)* **Total**	972 2 **974**	9.720.000 20.000 **9.740.000**

Dirt crawl space	#28	Blood Agar	35^3^	—	*Bacillus sp. * *Proteus sp* *Pseudomonas sp.*	TNC^4^	TNC^4^

Gravel, crawl space	#27	Blood Agar	35^3^	—	*Bacillus sp. * *Proteus sp.* *Pseudomonas sp.*	TNC^4^	TNC^4^

Sandal, under master bed	#36	Blood Agar	35^3^	—	*Bacillus sp. * *Proteus sp.* *Pseudomonas sp.*	TNC^4^	TNC^4^

**Table:** (b)

Endotoxins	Sample #	Sample type	Location	Concentration (EU/Swab)^5^
	#3	Swab	J-Tube, Under Sink	4930
	#4	Swab	Top, Kitchen Cabinet	24.800
	Blank	Swab	Field Blank	None Detected
	Blank	Swab	Lab Blank	None Detected

^1^These samples were tested to determine the major species of *Bacillus*.

^2^This sample was tested for *Actinomycetes* because of white mycelia type growth on wood truss.

^3^These samples were tested by RealTime Laboratories for the presence of bacteria species on samples tested for mycotoxins.

^4^CFU was not determined. TNTC: too numerous to count.

^5^Endotoxins were analyzed by ESML using LAL Kinetic Chromogenic Assay.

**Table 4:** This table summarizes the detection of trichothecenes, aflatoxins and ochratoxin A present in bulk samples taken from the master bath, master bedroom (sandal), and crawl space. The reported data are in ppb per mycotoxin.

Sample	Trichothecenes	Aflatoxins	Ochratoxin A
Towel—master bath	11.71	NP	4.9
Sandal—master bdrm	0.47	NP	3.4
Wood truss—crawl space	1.68	3.5	5.8
Gravel—crawl space	7.7	NP	7.7
Dirt—crawl space	2.1	NP	2.1
Plastic sheet—crawl space	NP	NP	2.8

Reported data are ppb.

NP: Not present.

Limit of Detection: Trichothecenes (0.2 ppb); Aflatoxins (1.0 ppb); Ochratoxin A (2.0 ppb).

**Table 5:** Mycotoxins present in body fluid of the five members of the family and the pet dog.

Patient specimen	Trichothecenes (ppb)	Aflatoxins (ppb)	Ochratoxin (ppb)
Father-Urine	NP	NP	18.2
Father-Nasal^1^ Secretion	NP	0.5 11.2	13 7.7
Mother-Urine	NP	NP	18.2
Mother-Nasal Secretion	1.02	1.2	1.6
Daughter-Urine	0.23	NP	28.0
Daughter-Nasal^2^ Secretion	4.68	NP	3.8
Son-Urine	0.2	NP	18.9
Son-Nasal Secretion	ND	ND	ND
Breast Milk	0.18	0.9	2.7
Placenta	NP	NP	4.2
Umbilical Cord	NP	NP	7
New Born-Urine	NP	NP	NP
Dog-Urine	1.49	NP	25.9
Dog-Ear Mass	23.07	0	2.2
Dog-Lipoma	20.9	0	1.4

Limits of Detection: Trichothecenes (0.2 ppb); Aflatoxins (1.0 ppb); Ochratoxin A (2.0 ppb).

ND: Not done.

NP: Not present.

^1^ *Pseudomonas aueroginosa* and *Penicillium* were cultured from the nasal secretions. These data represent two different tests.

^2^ *Acinetobacter sp.* was cultured from nasal secretion at too numerous to count. In addition, *Aspergillus fumigatus* was cultured from left ethmoid and sphenoid mucosal surgical specimen.
